# Recycled Concrete Aggregate for Medium-Quality Structural Concrete

**DOI:** 10.3390/ma14164612

**Published:** 2021-08-17

**Authors:** Dong Viet Phuong Tran, Abbas Allawi, Amjad Albayati, Thi Nguyen Cao, Ayman El-Zohairy, Yen Thi Hai Nguyen

**Affiliations:** 1 Department of Civil Engineering, Industrial University of Ho Chi Minh City, Ho Chi Minh City 70000, Vietnam; tranvietphuongdong@iuh.edu.vn (D.V.P.T.); nguyenthihaiyen@iuh.edu.vn (Y.T.H.N.); 2 Department of Civil Engineering, University of Baghdad, Baghdad 17001, Iraq; A.Allawi@uobaghdad.edu.iq (A.A.); A.khalil@uobaghdad.edu.iq (A.A.); 3 Faculty of Civil Engineering, Tien Giang University, Mỹ Tho 84000, Vietnam; caonguyenthi@tgu.edu.vn; 4 Department of Engineering and Technology, Texas A&M University-Commerce, Commerce, TX 75429, USA

**Keywords:** recycled coarse aggregate, recycled concrete, workability, ultrasound pulse velocity, durability, water absorption, abrasion

## Abstract

This paper reports an evaluation of the properties of medium-quality concrete incorporating recycled coarse aggregate (RCA). Concrete specimens were prepared with various percentages of the RCA (25%, 50%, 75%, and 100%). The workability, mechanical properties, and durability in terms of abrasion of cured concrete were examined at different ages. The results reveal insignificant differences between the recycled concrete (RC) and reference concrete in terms of the mechanical and durability-related measurements. Meanwhile, the workability of the RC reduced vastly since the replacement of the RCA reached 75% and 100%. The ultrasound pulse velocity (UPV) results greatly depend on the porosity of concrete and the RC exhibited higher porosity than that of the reference concrete, particularly at the transition zone between the RCA and the new paste. Therefore, the sound transmission in the RC required longer times than that in the reference concrete. Moreover, a predictive equation relating the compressive strength to the UPV was developed.

## 1. Introduction

As the global population grows at a fast rate, there is a great demand for concrete to develop infrastructure, urbanization, and accommodation, leading to the depletion of raw materials and natural aggregates for the production of concrete and cement manufacturing. Consequently, population growth raises concerns about greenhouse emissions and energy consumption issues. In addition to the depletion of natural aggregates and raw materials for contemporary constructions, the construction and demolition waste (CDW) existing in old structures is an imperative problem. The amount of CDW is approximately 850 million tons in the European Union, while it is 250 million tons in the USA [1,2]. The CDW is mainly landfilled or used as sub-base material, particularly in developing countries, making it a profound burden and a challenging task for environmental protection. Cutting down the exploitation of natural aggregates and reusing the CDW in the concrete industry are solutions to the problem. As soon as quality control of waste management was introduced and grew, a decrease in contamination was noticed [3]. The results show that the use of RCA from concrete significantly reduced the environmental impacts as well as the costs of the construction materials. Cement was the main contributor to both impacts [4]. Economic models were developed to show that the optimization of prices depends on: the price of demolition, transport costs, price of recycling, labor costs, and price of natural materials. The production of concrete with recycled aggregates was the most cost-effective in the case of parallel recycling of other materials and simultaneous clearing of the CDW [3].

There have been many attempts to produce RCA and recycled fine aggregate in the concrete industry. The properties of concrete containing ceramic waste aggregate and coal waste powder have been investigated [5,6]. These materials reduced greenhouses gases and showed the highest positive impact on the global warming index [5]. The red ceramics fine aggregate and artificial expanded clay coarse aggregate were used in the concrete mixture [7]. This paper analyses the surface resistivity (SR) and bulk resistivity (BR) of structural lightweight waste aggregate concrete (SLWAC) [7]. Recently, RCA was recommended for use in pavement construction (sub-base, anti-freeze layer, and sub-grade) and regular construction (as concrete, precast, and backfill), as well as for raising the ground level and for covering with soil [8]. Therefore, investigated the properties of medium-quality concrete incorporating RCA are investigated in this paper.

Lofty and Al-Fayez [1] found that replacement of the RCA by 10 to 30% and the recycled fine aggregate by 10 to 20% improved the mechanical properties and durability of concrete. The use of 50% RCA in recycled concrete (RC) provided similar durability as the reference concrete. Meanwhile, Andreu and Mirren [9] suggested 100% replacement of the natural coarse aggregate (NCA) by RCA to obtain high-performance concrete. However, the greater replacement of RCA by CDW led to higher adverse effects on concrete in terms of porosity and vulnerability [10,11,12,13,14]. The properties of concrete made using the CDW could be improved by using different techniques such as treating the CDW before using it [15,16,17], incorporating supplementary cementitious materials, or using nanosilica materials [18,19,20]. Moreover, the source and properties of the recycled materials can change the structural performance of concrete [21,22,23]. The RCA was produced from three different sources, including medium-strength concrete, high-performance concrete, and natural aggregate [23]. The results show that concrete with natural aggregates has a higher strength than that with the RC. Moreover, the recycled aggregate concrete at medium strength contains a loose and porous interfacial zone, while the dense interfacial zone was found in the high-performance concrete. The effect of the curing conditions on the performance of hardened concrete was studied in [24,25,26]. Notably, the RCA had a higher water demand than the natural aggregates, which led to reductions in the workability and durability of the RC.

The influence of the RCA on the RC properties was investigated in previous studies. However, more experimental investigations are still needed at different ages. Moreover, un-destructive tests must be conducted on the RC and evaluations of the compressive strength. Therefore, this paper reports evaluations of the mechanical properties and durability in terms of the abrasion of medium-quality concrete incorporating RCA at different ages. Concrete specimens were fabricated with various percentages of RCA (25%, 50%, 75%, and 100%).

## 2. Experimental Methodology

### 2.1. Materials

The specifications of the materials used in this study were tested by the authors. Ordinary Portland Cement (OPC), specified by ASTM C 150 [27], was used. Table 1 and Table 2 present the physical properties and chemical compositions of the used OPC, respectively.

**Figure 1:**
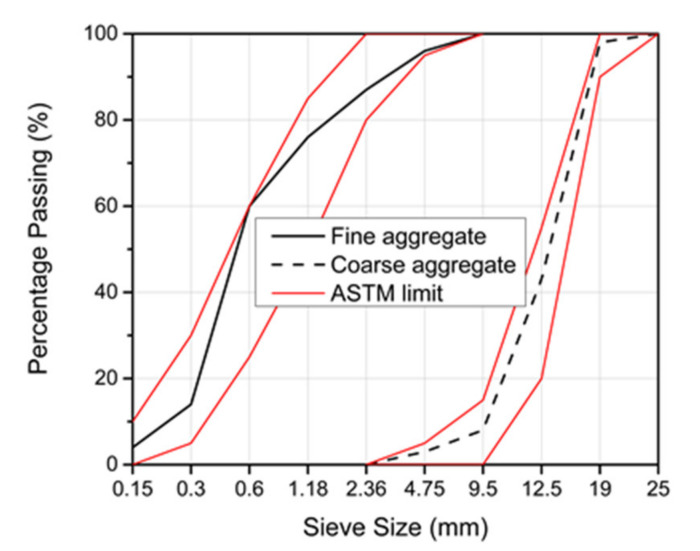
Grain size distribution of the fine and coarse aggregates.

All concrete mixtures used river sand as the fine aggregate, while RCA replaced the NCA either partially or fully. The used NCA was crushed river gravel with a grain size distribution, as shown in Figure 1. The specifications of the natural aggregates followed the requirements of standard ASTM C33 [28]. The physical properties of the natural aggregates, i.e., fineness modulus; specific gravity; bulk density; absorption; moisture content; sulphate content; and soundness, were determined by the authors according to [28] and are listed in Table 3 and Table 4, respectively.

**Figure 2:**
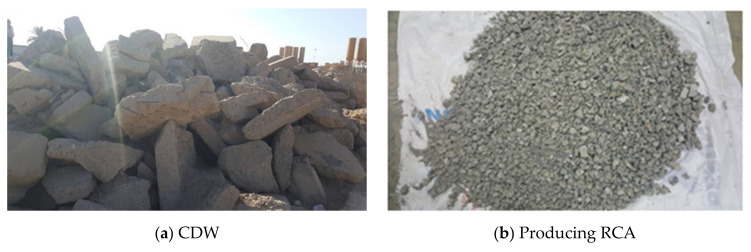
Recycling CDW as concrete aggregate.

The source of the used RCA was old reinforced concrete buildings and barriers, as shown in Figure 2. The RCA was prepared with a similar grading size distribution and maximum size to the NCA. The RCA showed lower unit weight and higher water absorption compared to those of the NCA (see Table 3).

### 2.2. Concrete Mixture

The laboratory program included the design of five concrete mixtures, as listed in Table 5. The concrete mixtures included a control concrete (CC) mix without RCA and four recycled concrete (RC) mixes with RCA replacements. The RCA content was varied as 25%, 50%, 75%, and 100% by volume. The concrete mix design followed the recommendations of the ACI 211.1 [29]. Moreover, the water to cement ratio was fixed at 0.45.

### 2.3. Preparation of Specimens

The concrete ingredients were mixed by a mechanical mixer for about 4 min until a homogenous mix was obtained. Concrete specimens were prepared according to ASTM C31/C31M [30]. The number of prepared specimens used in each test is listed in Table 6. In the curing process, all specimens were kept in the molds for 24 h before being subjected to a moist cure for 6 days. In the following 21 days, the specimens were kept in a controlled tank at room temperature and the relative humidity was kept at 50%.

### 2.4. Testing Methodology

All experiments were conducted in the Department of Civil Engineering Labs at Baghdad University, Iraq. Three specimens were tested for each group and the average results were adopted.

**Figure 3:**
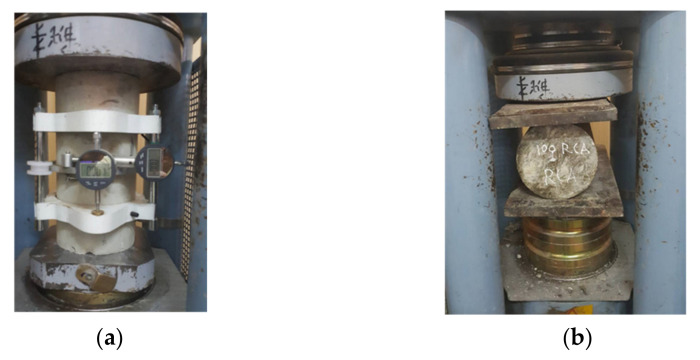
(**a**) Compression and modulus of elasticity tests, (**b**) splitting tensile tests.

A standard compression machine was used to conduct the compression and splitting tensile tests according to [31,32], respectively. This machine was working under a force-control with a loading rate of 9.0 kN/min (see Figure 3). The static modulus of elasticity was measured in accordance with ASTM-C469 [33]. Concrete cylinders with gauge lengths of 150 mm and dial gauges with a 0.002 mm accuracy were used to measure strains, which corresponded to 40% of the ultimate concrete strength (0.4 f_c_’), as shown in Figure 3a.

**Figure 4:**
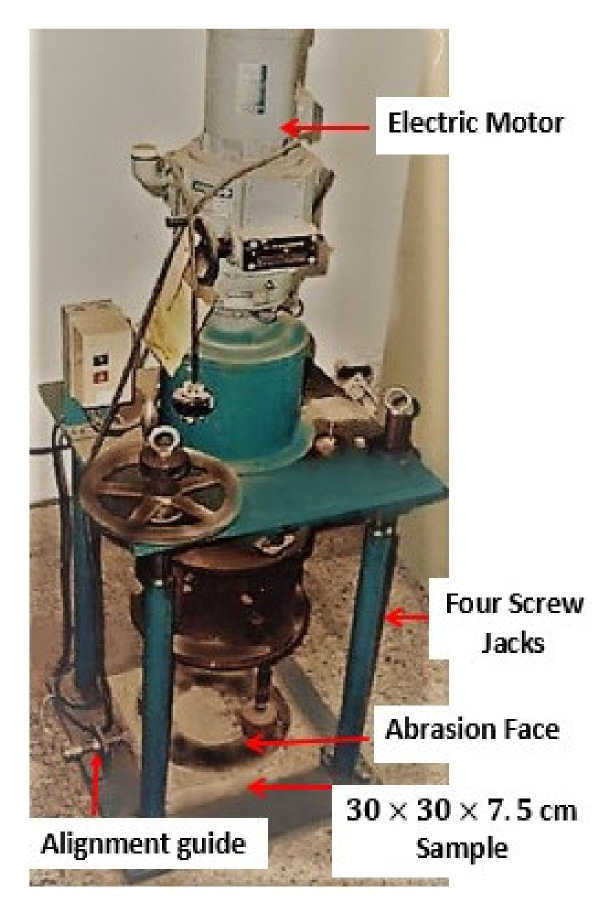
Revolving disk abrasion test machine.

The abrasion resistance of the RC surface was investigated using the standard test method according to [34]. The abrasion tests were performed using the Revolving disk abrasion test machine. In this method, friction forces were applied by rotating steel disks to grind and rub the concrete samples (see Figure 4). Height measurements of the specimens were taken throughout the test. The test period was 60 min so as to produce significant wear on the concrete faces.

**Figure 5:**
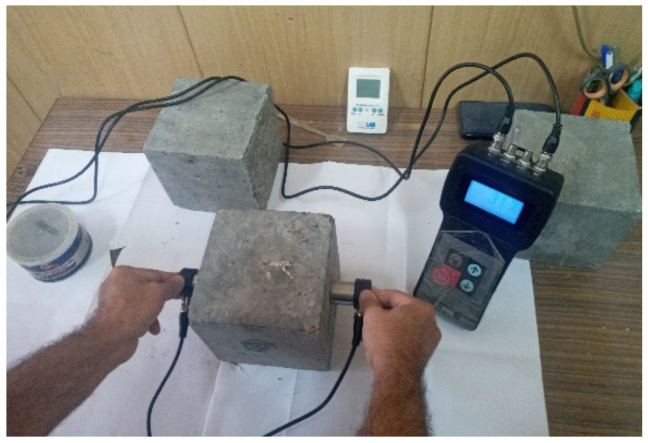
The ultrasound pulse velocity (UPV) tests.

Non-destructive testing of specimens at the age of 28-days was performed by passing an electronic wave through the concrete specimens according to [35]. Ultrasound pulse velocity (UPV) tests were conducted on the RC cubes (150 mm × 150 mm × 150 mm). Two 50 mm transducers rods were used in this method and positioned so that they were aligned at the same level opposite to each other (see Figure 5). The transducer properties were as follows: oscillation frequency of 54 kHz and transit time accuracy of ±1%. The results of the transmission times were measured and the ultrasonic wave velocity in concrete was obtained by dividing the transmission distance (150 mm) by the measured time. After that, the concrete cubes were tested under compression.

**Figure 6:**
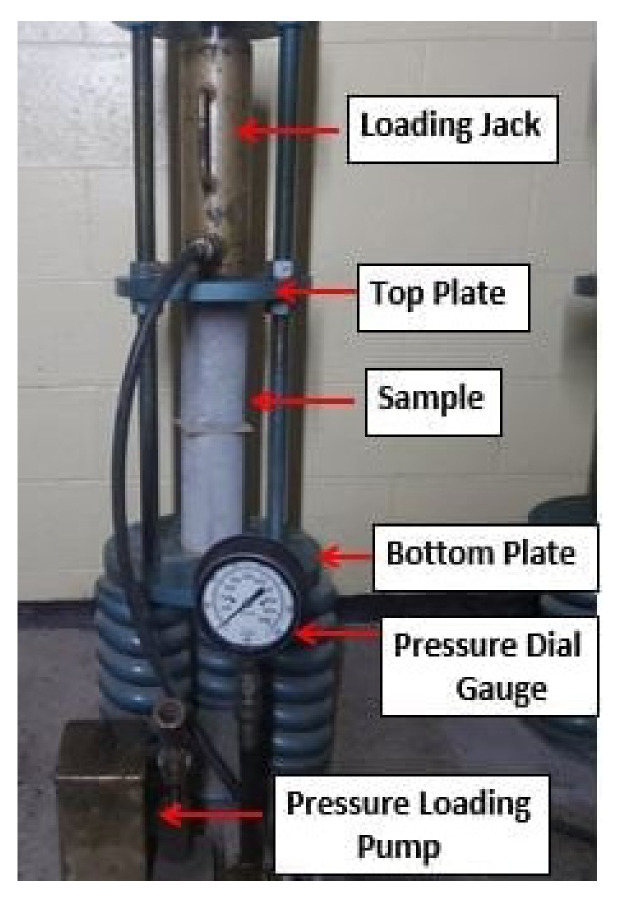
Creep test set up.

The RC specimens were exposed to sustained axial compressive loads to determine the effect of creep according to [36]. RC cylinders of 100 mm × 200 mm were used to investigate the effect of creep. The creep loading frame, as shown in Figure 6, was used to apply the sustained compressive load. The applied load was 40% of the compressive strength of the specimens. The compressive strain was measured using a dial gauge with an accuracy of 0.002 mm. This test was carried out at a conditional temperature of (21 ± 5 °C) and relative humidity of (35 ± 5%). The strain readings were taken after loading and then each week for the rest of the test. The load was modified periodically to avoid concrete relaxing strain. Finally, the load was released, and readings were taken accordingly.

**Figure 7:**
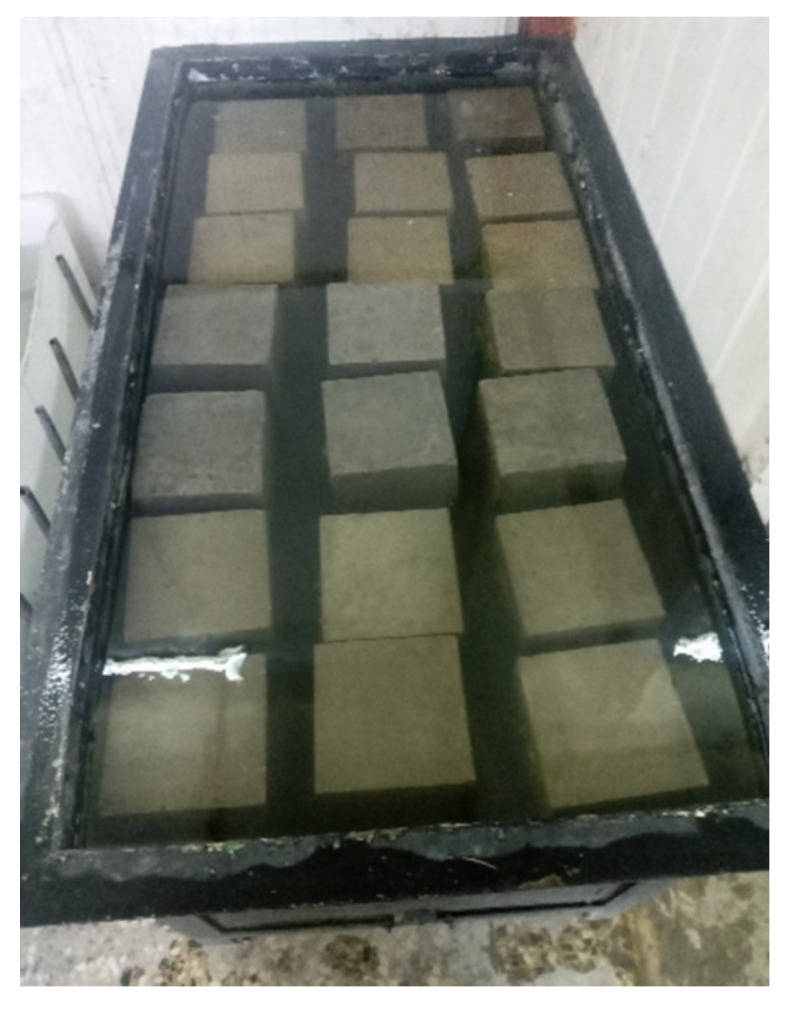
Specimens were immersed in the water tank for the absorption test.

The RCA influence on the RC density was examined according to [37]. Concrete cubes of 150 mm × 150 mm × 150 mm were poured and set for 24 h and then cured for a further 6 days underwater (see Figure 7). The density was determined as follows: 1—the specimens were weighed in air; 2—the specimens were weighed again underwater; 3—the difference in weights was the weight of water displaced; 4—the density was the weight in air divided by the weight of water displaced.

The influence of the content of the RCA on the water absorption of the RC was explored according to [37]. RC cubes of 150 mm × 150 mm × 150 mm were used. The specimens were oven-dried and then immersed in water. The increase in mass was measured as a percentage of the dry mass. The percentage of water absorption was calculated as follows:

Water absorption = [(B − A)/A] × 100%

where A was the mass of the oven-dried specimen, at temperature (100–110) °C for no less than 24 h and B was the mass of saturated specimens after immersion in water at approximately 21 °C for no less than 48 h.

## 3. Results and Discussions

The workability, mechanical properties, and durability in terms of the abrasion of cured concrete (compressive and tensile splitting strength, elastic modulus, creep strain, pulse velocity, abrasion resistance, density, and water absorption) were examined at different ages.

### 3.1. Workability

**Figure 8:**
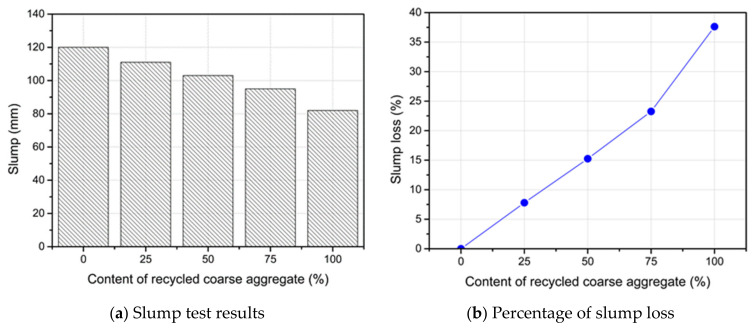
Workability of the RC mixtures with various contents of the RCA.

The workability of the fresh RC was examined using the slump flow test according to ASTM C143 [38]. Figure 8a demonstrates the results of the slump tests. The slump of the RC was lower than that of the CC mix. Inversely proportional relationships were found between the replacement content of the RCA and the slump test result. This low workability of RC is due to the hindrance in the movement of concrete particles by RCA particles. The higher surface area and water absorption of the RCA increased the need for water, which led to reductions in workability. The comparative analysis of slump has been performed using the percentage slump loss factor (Equation (1)).

(1) $$\mathrm{Slump}\;\mathrm{loss}\;\%=\frac{S_{o}-S_{f}}{S_{o}}\times 100\%$$

where S*_o_* is the slump in mm of the CC mix and S*_f_* is the slump in mm of the RC mix. The workability of concrete depends mainly on the water content and grading size distribution of the used aggregate. The effect of the grading size distribution can be minimized and eliminated in this study since the RCA was prepared so as to have the same distribution as the NCA. Therefore, the slump loss of the RC could be attributed to the effective water content that was left after absorbing into the capillary pores of the coarse aggregate. As presented in Table 4, the water absorption of the RCA was much higher than that of the NCA. Consequently, the effective water–cement ratio of the RC was low in comparison to the CC mix. This reduction in the effective water–cement ratio increased as the content of the RCA increased (see Figure 8b). The same trend was obtained when different contents of the RCA were used [39,40].

### 3.2. Compressive Strength

**Figure 9:**
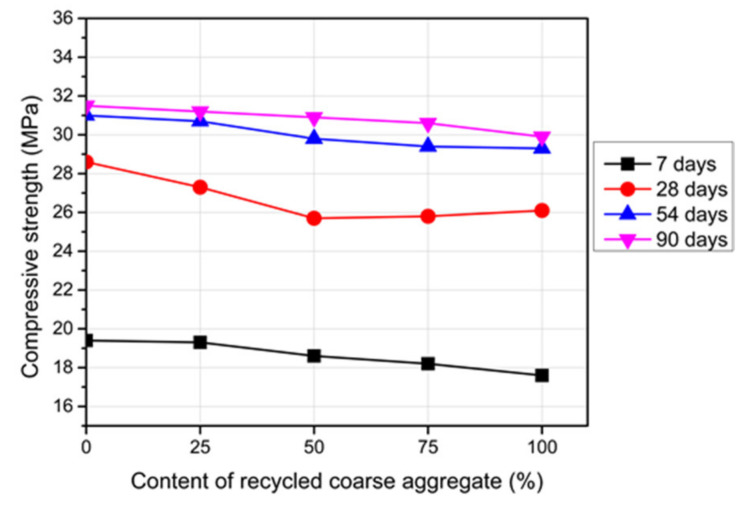
Compressive strength of the RC with different RCA contents.

The average compressive strength of the RC with various RCA contents is exhibited in Figure 9. The presented data are the average results of three concrete cylinders tested for each group at a certain age with a standard deviation range of 2.79% to 3.15%. The compressive strength was reduced in comparison to the reference concrete. More reductions in the compressive strength of the RC were obtained as the RCA replacements increased. These reductions were in the range of 5.0–9.3%. The existence of a weaker interfacial transition zone (ITZ) between the RCA and mortar, which is considered as the strength-limiting phase in concrete [41], was the main cause of these reductions in the compressive strength. The high water absorption of the RCA, which was gathered at the aggregate surface and resulted in more pores in the ITZ, contributed to form a weaker ITZ relative to the case of using NCA [42,43]. In addition, the low strength of the RCA relative to the NCA was another cause of the lower strength of the RC [44].

The failure shapes were well-formed cones for both ends for concrete with the NCA and RCA (25% and 50% replacements). Whereas, the failure mode was cone shapes at the bottom ends with vertical cracks for the other replacements.

Figure 9 exhibits the development of the compressive strength versus the concrete age. It shows clearly that the compressive strength of the reference concrete and RC increased with curing time, which increased at a rapid rate at an early age. However, this rate decreased after 28 days. Moreover, the early strength of the RC was about 65–80% of its 28-day compressive strength, while the reference concrete obtained only 56% of its strength at 28 days. The strength increase of the RC was higher than that of the reference concrete [9]. This difference in the development rate could be attributed to the late reaction of the residue of the non-hydrated cement on the surface of the RCA [45,46,47].

### 3.3. Splitting Tensile Strength

**Figure 10:**
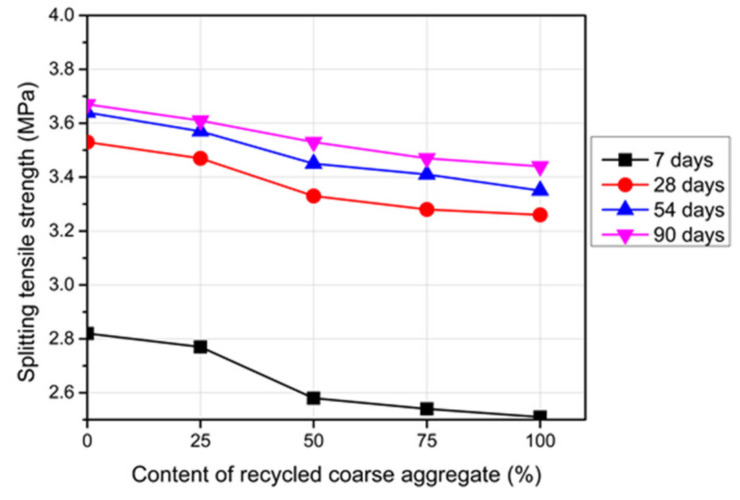
Splitting tensile strength of the RC with different RCA contents.

Figure 10 clarifies the experimental results of the splitting tensile strength of the reference concrete and RC at different ages. Using the RCA caused reductions in the splitting tensile strength. The higher content of RCA used, the lesser the tensile strength obtained. For 100% replacement with RCA, a 10.7% difference in the tensile strength was observed between the reference concrete and the RC at an age of 7 days. Whereas this reduction decreased to 8.3% for the age of 28 days and over. The residual cement mortar in the RCA provided weakened points, which caused reductions in the tensile strength of the RC. Previous studies confirmed 11–17% reductions in the splitting tensile strength due to 100% replacement of the RCA [39,47]. Moreover, the composition and the source of the RCA contributed significantly to these reductions.

### 3.4. Elastic Modulus

**Figure 11:**
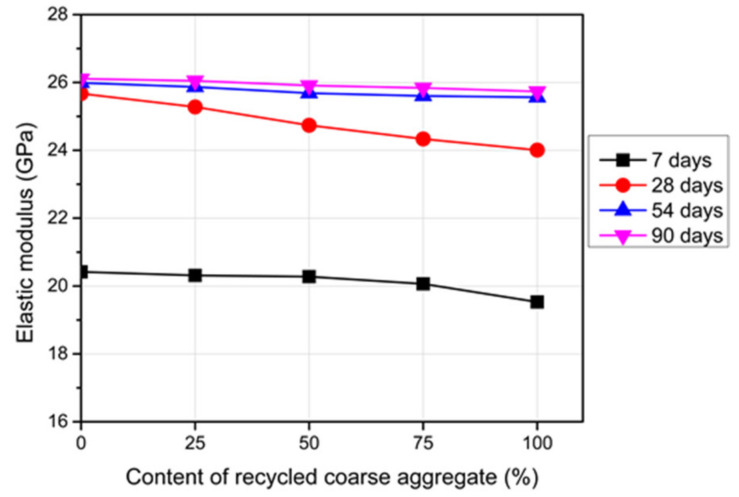
Elastic modulus of the RC with different RCA contents and ages.

The presented data are the average results of three concrete cylinders tested for each group at a certain age with a standard deviation range of 1.50% to 2.15%. The RC showed a lower elastic modulus relative to the reference concrete, as presented in Figure 11. The difference in the elastic modulus between the RC and the reference concrete was 7.7% for a 100% RCA content after curing for 28 days; the reduction in the elastic modulus became higher when the percentage of the RCA increased. The relative lower stiffness as well as the higher porosity of the RCA were the main reasons for this reduction.

The difference in the elastic modulus between the reference concrete and the RC in this study was lower than that observed in previous studies [2,9,45]. This difference could be attributed to the various sources of the RCA as well as the quality control during the preparation and casting processes.

### 3.5. Abrasion

**Figure 12:**
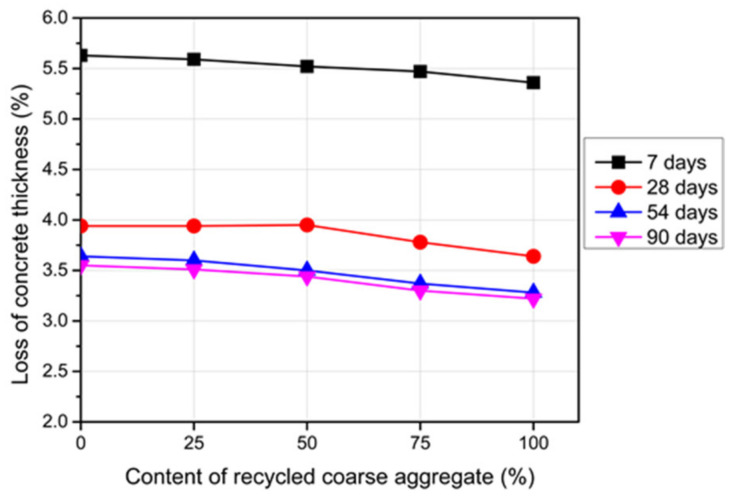
Abrasion resistance of the RC with different RCA contents and ages.

Mechanical degradation of concrete due to vehicular movement or pedestrian traffic causes loss of concrete mass, which is defined as abrasion. Figure 12 shows the results of the abrasion resistance of the RC and reference concrete in terms of the concrete thickness loss at different ages. The results confirm that the abrasion wear rate of the RC was lower relative to the reference concrete. This means that a higher abrasive wear resistance was obtained and this resistance was enhanced as the RCA content increased. This phenomenon could be attributed to the roughness of the RCA due to the long-adhered cement paste. The formed good bond between the new paste and the mortar of the RCA improved the abrasion resistance of the RC [48]. Moreover, the type of RCA could affect this resistance [44].

The abrasion wear of the RC and reference concrete increased at an early age, which led to reductions in the concrete thickness. Notably, the concrete thickness loss was significantly high at the age of 7 days—the specimens lost approximately 5.0 mm of the thickness (see Figure 12). However, the thickness loss was insignificant after the age of 28 days.

### 3.6. Ultra-Sonic Pulse Velocity

**Figure 13:**
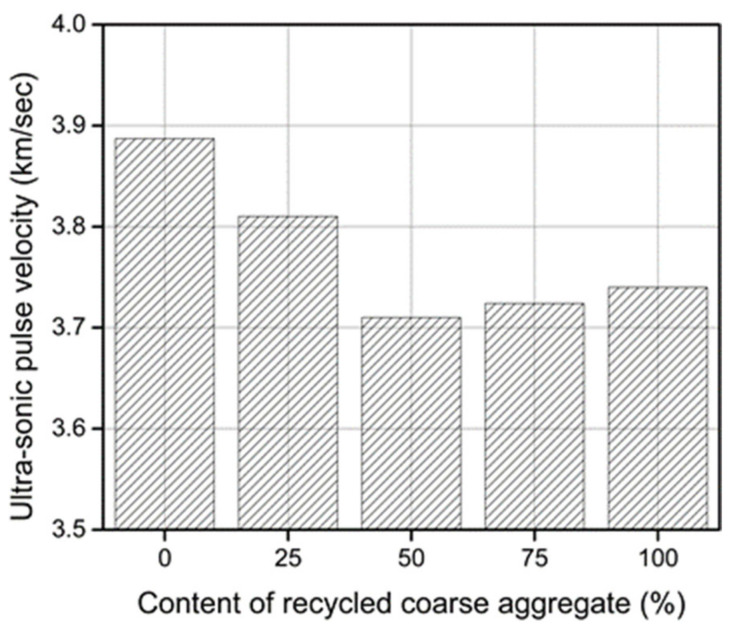
Ultra-sonic pulse velocity of concrete with various contents of the recycled aggregate at the age of 28 days.

The measured results of the ultrasound pulse velocity (UPV) tests are shown in Figure 13. The UPV values decreased with the incorporation of the RCA in concrete. The UPV values greatly depend on the porosity of concrete and the RC exhibited higher porosity than that of the reference concrete, particularly at the transition zone between the RCA and the new paste. Therefore, the sound transmission in the RC required longer times than that in the reference concrete.

**Figure 14:**
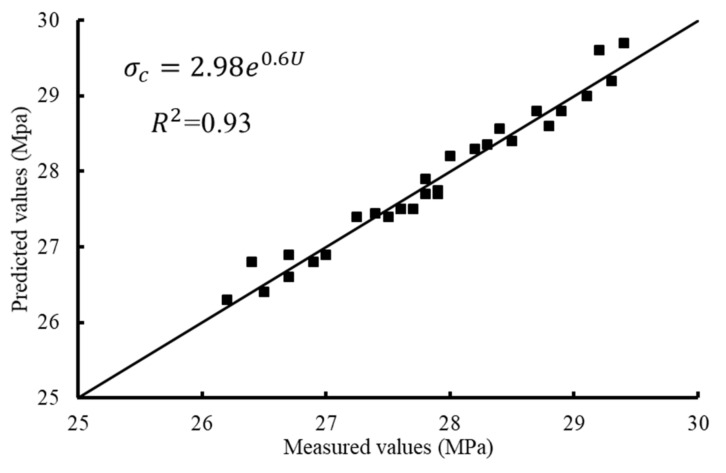
Regression between the predicted and measured values of the compressive strength.

A regression analysis was conducted to develop a predictive equation that relates a criterion variable to a predictor variable. The regression analysis is useful to estimate the compressive strength of the recycled concrete using a non-destructive test (UPV test). In this research, the criterion variable included the compressive strength at 28 days and the predictor variable was the ultrasonic wave velocity. Two approaches were used to assess the adequacy of the proposed statistical regression model. The first approach was based on examining goodness of fit measures and the coefficient of multiple determinations (R^2^). Whereas, the second approach was based on the graphical analysis of the residuals (diagnostic plots). The Minitab statistical software V. 17 was used for the model development. Two groups of experimental results were used for this target. The first group consisted of the 30 data points that represent the original test results. The second group included an additional 15 data points (additional compressive strength tests) to be adopted for the proposed model justification. The model equation was as follows.

(2) $$\sigma_{c}=2.98\;e^{0.6U}$$

where σ_c_ is the compressive strength at the age of 28 days in MPa and U is the UPV (km/s100). The validation process to assess the adequacy of the proposed prediction model and measure the error or accuracy of the prediction is exhibited in Figure 14.

### 3.7. Effect of Creep

**Figure 15:**
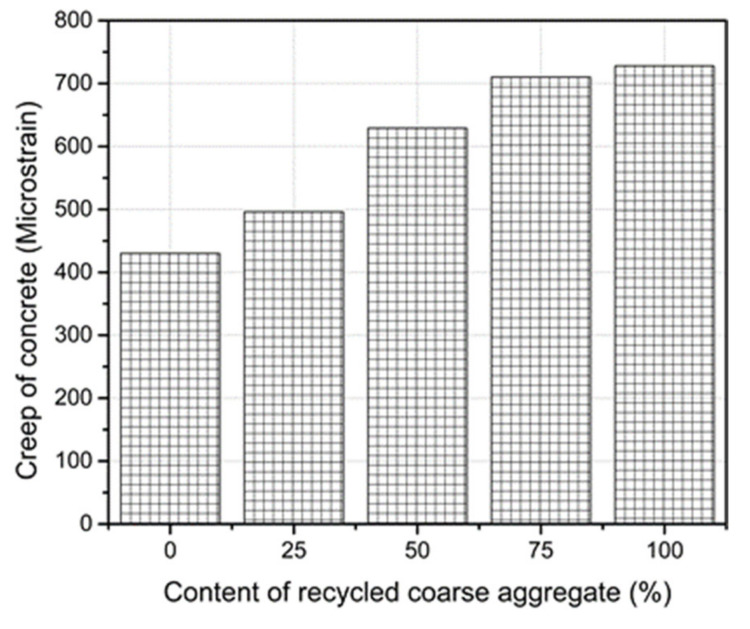
Creep strains of the RC with various RCA contents.

The creep tests were conducted for 90 days and the recorded creep strains are presented in Figure 15. The creep strain of the RC increased as the RCA content increased. For 100% replacement of the RCA, the creep strain increased by 67.5% relative to the reference concrete. However, only a 16% increase was obtained for the case of 25% replacement. The higher volume of cement paste in the RC compared to that of the reference concrete caused higher losses of the physically absorbed water under sustained stresses and, subsequently, led to the increase in creep strains. The adverse effects of using RCA on the concrete creep mainly include the increase in beam deflection and the reduction in the structural bearing capacity. In addition, the increase in the creep of the RC can cause excessive prestressing losses of the prestressed members [49,50,51].

### 3.8. Density of the RC

**Figure 16:**
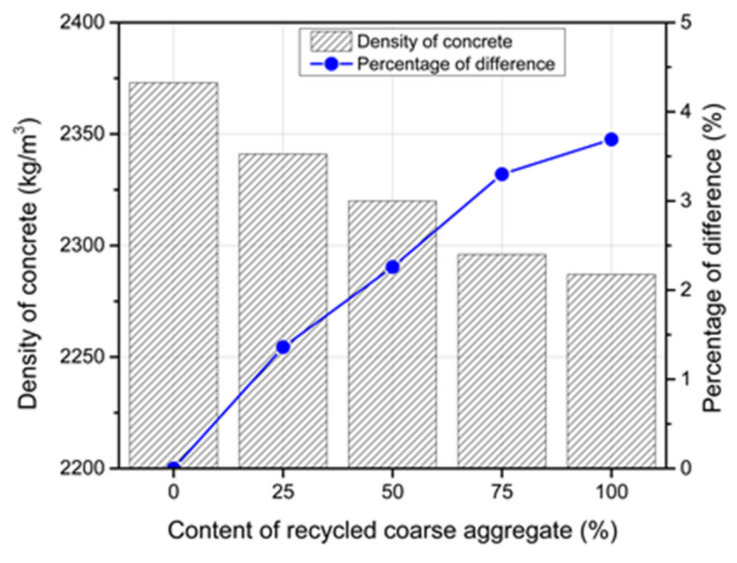
Variations in the recycled concrete density at the age of 28 days.

The lower unit weight of the RCA relative to the NCA led to reductions in the RC density and these reductions increased as the RCA content increased (see Figure 16). For the RCA content of 100%, a reduction of 3.9% was obtained. This reduction was reported as 3.3% by Etxeberria et al. [52] and 5% by Verian [53] for the same RCA content. The adhered mortar in the RCA was lightweight compared to the NCA of the same volume, which caused the decrease in density [54].

### 3.9. Water Absorption

**Figure 17:**
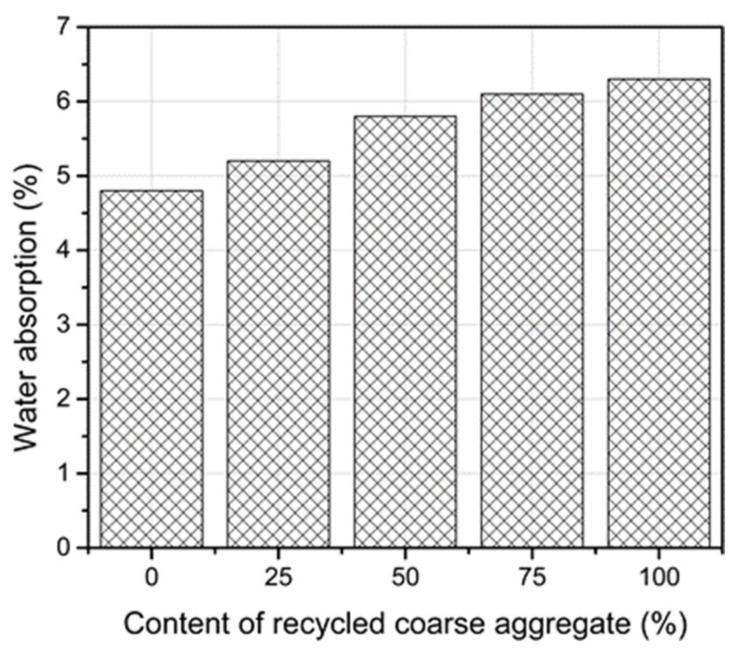
Water absorption of the RC with various contents of the RCA.

Water absorption of concrete can be an indirect indication of concrete durability. Absorbed water carries aggressive ions such as sulfate, chloride, and hydrogen ions into concrete, which cause reactions with the cement paste and affect the concrete durability. Figure 17 presents the variation in the water absorption of the RC with various contents of the RCA. Due to greater water absorption of the RCA compared to the NCA (see Table 4), using the RCA increased the water absorption of the RC. A linear relationship was observed between the water absorption and the content of the RCA. For 100% replacement of the RCA, the water absorption increased by approximately 7%. Previous studies confirmed the same increase in water absorption [2,9]. The increase in the water absorption of the RC is a problem for the industry because the more water absorbed into the concrete, the easier movement of ions from external exposure into concrete is. Consequently, the corrosion resistance of the reinforced RC is affected.

### 3.10. Microstructural Analysis

**Figure 18:**
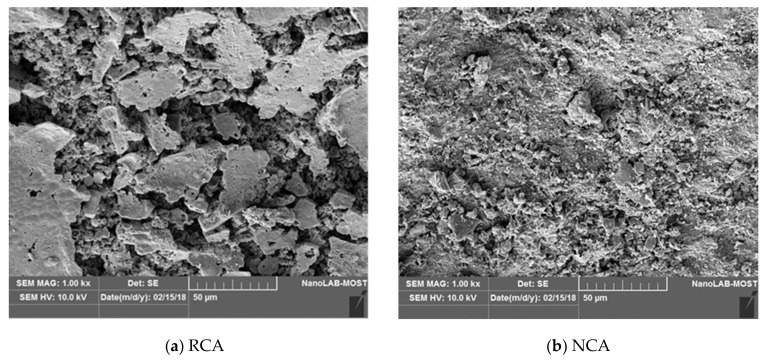
Microscopic analysis of the (**a**) RCA and the (**b**) NCA.

Figure 18 demonstrates the results of the microscopic analysis for the RCA and NCA. The good bond between the NCA and cement paste produced a much more refined surface for the reference concrete. Whereas, the RCA formed pores and roughness on the RC surface, where water accumulated and formed porous and weak ITZ. This difference led to reductions in the mechanical properties of the RC relative to the reference concrete.

## 4. Discussion on the Practical Aspects of Using RC

The ACI 318-19 code [55] specified that 17 MPa is the minimum compressive strength requirement for concrete used in structural members. As a consequence of the experimental test results in terms of the compressive strength and density, the RCA produced from old reinforced concrete buildings and barriers can be used to produce RC, which can be used as structural concrete in the field of construction. However, reductions in elastic modulus and increase in creep deformations should be considered in the design of the long-term behavior. Moreover, additional protections to the RC surface should be provided against the aggressive environment and vehicular or pedestrian traffic to protect the steel reinforcement against corrosion due to the increase in the water absorption and abrasion wear rate.

## 5. Conclusions

This paper reports an evaluation of the properties of medium-quality concrete incorporating RCA. Concrete specimens were poured with various contents of the RCA (25%, 50%, 75%, and 100%). The workability, mechanical properties, and durability in term of abrasion of cured concrete were examined at different ages. According to the experimental results, the following findings were concluded.

1. The high demand for water of the RCA significantly affected the workability as well as the durability of the RC. The workability of the RC was reduced by 8% to 38%.
2. The compressive strength was reduced by 5.0–9.3%, in comparison to the reference concrete. More reductions in the compressive strength of the RC were obtained as the RCA replacements increased.
3. For 100% replacement of the RCA, a 10.7% difference of the tensile strength was observed between the reference concrete and the RC at the age of 7 days. Whereas, this reduction decreased to 8.3% at an age of 28 days and over. The residual cement mortar in the RCA provided weakened points, which caused reductions in the tensile strength of the RC.
4. The concrete thickness loss was significantly high at an early age (age of 7 days), where the thickness of the specimens decreased by approximately 5.0 mm. However, the thickness loss was insignificant after 28 days.
5. The provided regression analysis is useful to estimate the compressive strength of the recycled concrete using a non-destructive test with a goodness of fit of 0.93.
6. For 100% replacement of the RCA, the creep strain increased by 67.5% relative to the reference concrete. However, only a 16% increase was obtained for the case of 25% replacement. The higher volume of cement paste in the RC compared to that of the reference concrete caused higher losses of the physically absorbed water under the sustained stresses and, subsequently, led to this increase in the creep strains.
7. Using the RCA as a replacement for the NCA led to an increase in the water absorption of the RC and a linear proportion was observed between the content of the RCA and water absorption. For 100% replacement of the RCA, the water absorption increased by approximately 7%.

## Figures and Tables

**Table 1:** Physical properties of OPC.

Properties		Result
Fineness by air permeability method (Blain)		296 m^2^/kg
Initial Setting Time		70 min
Final setting time		310 min
Soundness (Autoclave Method)		0.4%
Compressive Strength	3-day age 7-day age	21.3 MPa 29.4 MPa

**Table 2:** Chemical components of the OPC (unit: %).

Oxides							Compound Composition			
CaO	SiO_2_	Al_2_O_3_	MgO	Fe_2_O_3_	SO_3_	LOI	C_3_S	C_2_S	C_3_A	C_4_AF
63.25	19.57	6.1	3.4	3.68	2.73	2.36	56.21	13.83	9.94	11.18

**Table 3:** Properties of the natural fine aggregate.

Properties	Values
Fineness Modulus	2.62
Apparent Specific Gravity	2.65
Bulk Density (kg/m^3^)	1773
Water absorption (%)	0.83
Moisture Content (%)	0.32
Sulphate Content, SO_3_ (%)	0.3

**Table 4:** Properties of the NCA and RCA.

Properties	NCA	RCA
Bulk specific gravity	2.632	2.231
Water absorption (%)	0.26	2.91
Soundness (MgSO_4_)	5.7	7.8

**Table 5:** The concrete mix ratios.

Materials	Weight (kg/m^3^)				
	CC	RC25	RC50	RC75	RC100
Water	189	189	189	189	189
Cement	420	420	420	420	420
Natural fine aggregate	753	753	753	753	753
Natural coarse aggregate	976	732	488	244	-
Recycled coarse aggregate	-	244	488	732	976

Remarks: CC is the control concrete mix, RCx is the recycled concrete with “x” percentage of replacement of the RCA, i.e., 25%, 50%, 75%, and 100%.

**Table 6:** Prepared specimens assigned to each test.

Specimen Shape	Number of Specimens	Type of Test
Concrete cylinders 150 mm × 300 mm	120	60 specimens for the compression tests. 60 specimens for the splitting tension tests.
Concrete cylinders 100 mm × 200 mm		15 specimens for the creep tests.
Concrete cubes 150 mm × 150 m × 150 mm	45	15 specimens for the ultrasound pulse velocity tests. 15 specimens for the density tests.15 specimens for the water absorption test.
Concrete Cuboids 300 mm × 300 mm × 75 mm	60	60 Specimens for the abrasion tests.

## Data Availability

The data presented in this study are available on request from the corresponding author.
